# Vibrational Corrections to NMR Spin–Spin Coupling Constants from Relativistic Four-Component DFT Calculations

**DOI:** 10.1021/acs.jpca.2c05019

**Published:** 2022-09-22

**Authors:** Katarzyna Jakubowska, Magdalena Pecul, Kenneth Ruud

**Affiliations:** † Faculty of Chemistry, University of Warsaw, 02-093 Warsaw, Poland; ‡ Hylleraas Centre for Quantum Molecular Sciences, Department of Chemistry, UiT—The Arctic University of Norway, N-9037 Tromsø, Norway; § Norwegian Defence Research Establishment, P.O. Box 25, 2027 Kjeller, Norway

## Abstract

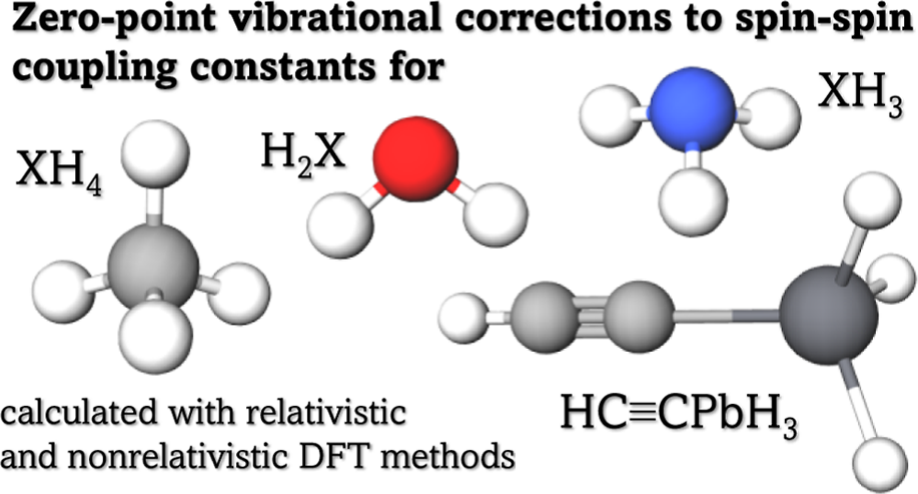

Zero-point vibrational (ZPV) corrections to the nuclear spin–spin coupling constants have been calculated using four-component Dirac–Kohn–Sham DFT for H_2_X (where X = O, S, Se, Te, Po), XH_3_ (where X = N, P, As, Sb, Bi), and XH_4_ (where X = C, Si, Ge, Sn, and Pb) molecules and for HC≡CPbH_3_. The main goal was to study the influence of relativistic effects on the ZPV corrections and thus results calculated at relativistic and nonrelativistic approaches have been compared. The effects of relativity become notable for the ZPV corrections to the spin–spin coupling constants for compounds with lighter elements (selenium and germanium) than for the spin–spin coupling constants themselves. In the case of molecules containing heavier atoms, for instance BiH_3_ and PbH_4_, relativistic effects play a crucial role on the results and approximating ZPV corrections by the nonrelativistic results may lead to larger errors than omitting ZPV corrections altogether.

## Introduction

The standard approach to calculations of molecular properties within the Born–Oppenheimer approximation is to evaluate them at some reference geometry, usually the equilibrium geometry. However, it is well known that high-precision calculations of molecular properties require taking into account vibrational corrections.^1,2^ This is particularly true of the NMR properties: nuclear spin–spin coupling constants and nuclear shielding constants, which both are sensitive to geometry distortions and thus to effects associated with nuclear motion.

There are several approaches for evaluating vibrational corrections to the spin–spin coupling constants,^3−5^ differing in accuracy and computational cost. The majority of the effect can be approximated by computing the zero-point vibrational (ZPV) corrections,^5^ that is, the difference between the equilibrium value and the averaged value for the ground vibrational state. ZPV corrections are usually calculated by perturbation theory^6−8^ and included in accurate computational studies.

On the other hand, it is well known that relativistic effects (understood as a difference between the results obtained using relativistic and nonrelativistic Hamiltonians) on NMR parameters can be non-negligible already for third-row elements.^9^ When both relativistic and vibrational corrections need to be accounted for, it is usually done by an incremental approach: calculating zero-point vibrational corrections using a nonrelativistic Hamiltonian and adding them to the relativistic value. This assumes that the nonrelativistic property and energy surfaces are sufficiently close to being parallel to the correct relativistic ones, or, in other words, that the relativistic corrections are similar for all geometries close to the equilibrium geometry. For many systems, this approach has been applied successfully^10,11^ but it is not always the case: it has been shown that in some cases^12^ derivatives of the spin–spin coupling constants with respect to internuclear distance can even differ in sign when calculated with nonrelativistic and relativistic Hamiltonians. There is, therefore, a need to calculate also ZPV corrections at the relativistic level of theory in order ensure correct estimates for these effects.

## Methods

### Theory

The most popular approach to calculating vibrational corrections to NMR parameters is the approach of Kern et al.,^6−8^ in which second-order perturbation theory is used. It has also been applied in the present work. It should be noted that this method implies only small-amplitude nuclear motions. In the case of large-amplitude nuclear motions (e.g., internal rotation) other methods, for example, molecular dynamics, must be employed,^13−15^ as it is important to distinguish conformational equilibria from large-amplitude motions.

In the perturbational approach, the unperturbed ground-state vibrational wavefunction is written as a product of harmonic oscillator wavefunctions in normal coordinates^5,16^
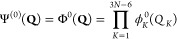
1where ϕ_*K*_^*n*^(*Q*_*K*_) is the *n*th excited harmonic oscillator state of the Kth normal vibrational mode, and the summation runs over 3*N* – 6 normal modes, where *N* is the number of atoms in the molecule. In the next step, a full set of virtual excitations from Ψ^(0)^(**Q**) is used to expand the first-order correction to the ground-state vibrational wavefunction, Ψ^(1)^(**Q**). If the formula for Ψ^(1)^(**Q**) is limited to the third-order Taylor expansion of the potential energy surface, the only relevant contributions are from single and triple excitations Φ_*K*_^1^(**Q**) and Φ_*K*,*L*,*M*_^3^(**Q**)(*K* + *L* + *M* = 3)
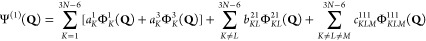
2

Here, for example, Φ_*KLM*_^*ABC*^(**Q**) has been obtained from Φ^0^(**Q**) by exciting the *K*th, *L*th, and *M*th modes to the Ath, Bth, and Cth harmonic oscillator states, respectively. The expansion coefficients in the above can be written (in atomic units) as^17^
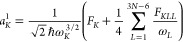
3
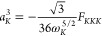
4
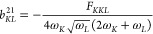
5
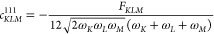
6where
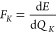
7
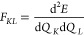
8
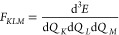
9and ω_*K*_ is the mass-weighted harmonic frequency for the *K*th normal mode. In the equilibrium geometry *F*_*K*_ = 0.

A vibrationally averaged molecular property *P* can be now calculated as an expectation value
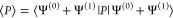
10

If *P* is expanded in a Taylor series about the equilibrium geometry
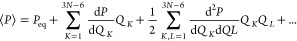
11combining eqs 1, 2, 10, and 11 and collecting terms of the same order gives
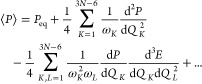
12

The final form of the formula for the ZPV correction to a property *P* is therefore
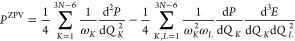
13

The first term in the above equation is the harmonic contribution to the ZPV correction and the second term is the anharmonic contribution.

This formula has been used in the present work to calculate ZPV corrections to the nuclear spin–spin coupling constants.

### Implementation

Our program works as an external driver to the Dirac^18^ program package, but can in principle be adapted to any other program. The ZPV corrections to the spin–spin coupling constants are calculated with the approach of Kern et al.^6−8^ using eq 13. In the case of NMR parameters, there is no analytic implementation for the energy and property derivatives and thus the method is fully numerical, which means that the first and diagonal second derivatives of the spin–spin coupling constants, as well as the harmonic frequencies and the semi-diagonal part of the cubic force field, are calculated numerically.

### Numerical Derivatives

The molecular Hessian, normal coordinates, and vibrational frequencies are calculated as described in our previous paper.^19^ Once the vibrational frequencies and normal coordinates are computed, the first and second derivatives of the spin–spin coupling constants with respect to geometric distortions along the normal coordinates of the molecule are calculated using three-point formulas^20^
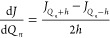
14
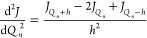
15

The semi-diagonal part of the cubic force field is calculated in the same fashion^20^
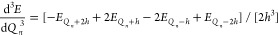
16
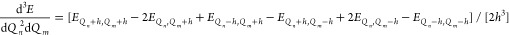
17

The approach thus involves performing a number of energy and property calculations, in which atoms are being displaced from their original positions along the normal coordinates. In the case of a nonlinear N-atom molecule (with 3N-6 vibrational modes), 45*N*^2^ – 165*N* + 150 single-energy computations and 6*N* – 11 property computations need to be run to determine the ZPV corrections.

When carrying out numerical differentiation, it is essential that an appropriate step length (*h* in the above equation) is used to ensure numerically accurate results. On one hand, if the step length is too small, numerical errors will dominate due to the approximate solution of the perturbed wavefunctions. On the other hand, if it is too large, the derivatives will be contaminated by higher-order terms. We have performed test calculations of the ZPV correction for the water molecule with a number of different step lengths in the range of . The calculations turned out to be numerically stable for step lengths between . Based on the above, for all subsequent calculations we have used a step length of .

## Computational Details

### Geometry Optimization

Geometry optimizations have been performed using the Dirac^18^ program at the same level of theory as the ZPV correction calculations carried out afterward in order to ensure that the molecular gradient is zero (a condition for the harmonic approximation). The convergence threshold for the gradient was 10^–4^ au.

### Single-Point Energy and Property Calculations

The four-component Dirac–Kohn–Sham energy and property calculations have been carried out with the Dirac^18^ program. Unless stated otherwise, the uncontracted aug-cc-pVTZ basis set^21^ on the hydrogen atoms and Dyall’s uncontracted triple-ζ basis set^22−24^ (dyall.v3z) on all the other atoms have been applied together with the B3LYP^25−28^ exchange–correlation functional.

For comparison, also nonrelativistic calculations have been carried out. In the case of the nonrelativistic computations, the speed of light has been scaled to 2000.0 au in the Dirac–Coulomb Hamiltonian.

Because the semi-diagonal part of the cubic force field was calculated numerically, the convergence threshold for all the single-point energy calculations needed to be tight. For this reason, the convergence threshold for the error vector was set to be 10^–10^ and in a few cases (about 10%) 10^–8^ if the number of iterations exceeded 50.

### Molecules under Investigation

In order to test the newly developed method for calculating ZPV corrections to spin–spin coupling constants, simple systems consisting of 3, 4, and 5 atoms have been chosen:

- H_2_X where X = O, S, Se, Te, Po;
- XH_3_ where X = N, P, As, Sb, Bi; and
- XH_4_ where X = C, Si, Sn, and Pb.

For some of these systems, vibrational corrections to the nuclear spin–spin coupling constants are known in the literature.^29−31^

In addition to this, to illustrate the usefulness of the method for larger systems, we have calculated ZPV corrections to the spin–spin coupling constants for an acetylene derivative, HC≡CPbH_3_.

As the vibrational frequencies are incorporated in the formula for the ZPV correction (see eq 13) and vibrational frequencies change for different isotopes of the same element, we needed to select the isotopic constitution of the molecules for which the calculations were performed. In the case of J(H–X) couplings, ^1^H and the most abundant magnetic isotopes of element X (^17^O, ^33^S, ^77^Se, ^125^Te, ^209^Po, ^14^N, ^31^P, ^35^As, ^123^Sb, ^209^Bi, ^13^C, ^29^Si, ^73^Ge, ^119^Sn, and ^207^Pb) were chosen (although we are aware that for many of them, the measurements of the spin–spin coupling constants are not possible because of the quadrupole moment of the nucleus and thus the associated line broadening). In the case of J(H–H) couplings, the computations were carried out for ^1^H and the most abundant isotope of element X: ^16^O, ^32^S, ^80^Se, ^130^Te, ^209^Po, ^14^N, ^31^P, ^35^As, ^121^Sb, ^209^Bi, ^12^C, ^28^Si, ^74^Ge, ^120^Sn, and ^207^Pb. As far as the HC ≡CPbH_3_ molecule is concerned, in order to limit the computational cost, the calculations were run only for ^1^H, ^13^C, and ^207^Pb.

## Results and Discussion

### Spin–Spin Coupling Constants

Even though the main focus of this work is to analyze the role that relativistic effects play on the ZPV corrections to spin–spin coupling constants, the results for the spin–spin coupling constants themselves will be briefly discussed for the sake of completeness. They have been collected in Table 1.

**Table 1:** Spin–Spin Coupling Constants, *J* [Hz], and Reduced Spin–Spin Coupling Constants, *K* [10^19^·m^–2^·kg·s^–2^·Å^–2^], for H_2_X, XH_3_, and XH_4_ Systems Calculated with Relativistic and Nonrelativistic Methods^a^

	^2^*J*_HH_		^1^*K*_XH_	
	nrel	rel	nrel	rel
H_2_O	–4.8	–4.9	42.5	42.6
H_2_S	–10.1	–10.1	23.4	23.4
H_2_Se	–10.0	–9.7	8.9	9.0
H_2_Te	–9.2	–8.8	9.6	–42.0
H_2_Po	–8.9	–7.3	10.7	–446.5
NH_3_	–6.8	–7.5	47.4	48.4
PH_3_	–10.5	–10.9	30.7	30.4
AsH_3_	–10.2	–10.7	14.9	15.0
SbH_3_	–10.0	–9.8	39.5	–11.5
BiH_3_	–8.5	–14.2	41.9	–462.4
CH_4_	–9.9	–10.7	39.4	39.6
SiH_4_	3.4	3.1	80.0	81.3
GeH_4_	9.0	9.1	218.6	218.8
SnH_4_	11.4	16.3	307.4	403.2
PbH_4_	15.4	38.0	403.0	1077.3

a Functional: B3LYP, basis set: aug-cc-pVTZ (on H) + dyall.v3z (on X).

In the case of couplings that involve the X atoms, which have different magnetogyric constants, we discuss reduced spin–spin coupling constants, *K*, due to their independence with the magnetogyric constants. Relativistic effects are noticeable and relevant in the case of ^1^*K*_XH_ for H_2_Te, H_2_Po, SbH_3_, BiH_3_, SnH_4_, and PbH_4_. For H_2_Te, SbH_3_, and BiH_3_, a change in the method from nonrelativistic to relativistic leads to changes in the absolute values of the coupling constants by an order of magnitude as well as a change in its sign. As far as H_2_Po is concerned, in addition to the change in sign, the absolute values of the coupling constants change by 2 orders of magnitude. Already in the case of SnH_4_, the relativistic effects constitute about 31% of the value calculated with the nonrelativistic method, and in the case of PbH_4_, it is 147%, which means that the nonrelativistic value is unable to provide even a qualitative estimate of the coupling constant value.

As far as ^2^*J*_HH_ is concerned, an effect analogous to the HALA effect^32−34^ is significant and cannot be neglected for H_2_Po, BiH_3_, SnH_4_, and PbH_4_. In the case of H_2_Po, it causes a decrease in the absolute value of the spin–spin coupling constant by 18% and in the case of BiH_3_, SnH_4_, and PbH_4_ it causes an increase by 67, 43, and 146%, respectively.

All of the above findings are in line with previous studies.^35−38^

### Effects of Relativity on the First and Second Derivatives of Spin–Spin Coupling Constants

The ZPV corrections to the spin–spin coupling constants depend on the first and second derivatives of the coupling constants with respect to nuclear distortions, the cubic force field, and the harmonic vibrational frequencies. Each of these parameters can to a different extent be sensitive to relativistic effects. We have, therefore, also investigated the influence of relativity on the first and second derivatives of the coupling constants with respect to normal coordinates. The results calculated with the relativistic and nonrelativistic approaches are shown in Table 2, for the sake of brevity only for the H_2_X systems. All the following observations can be generalized to the XH_3_ and XH_4_ systems.

**Table 2:** First  and Second  Derivatives of Spin–Spin Coupling Constants with Respect to Normal Coordinates for H_2_X Systems Calculated with Relativistic and Nonrelativistic Methods^a^

		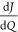				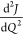			
		^1^*J*_XH_		^2^*J*_HH_		^1^*J*_XH_		^2^*J*_HH_	
		nrel	Rel	nrel	rel	nrel	rel	nrel	rel
H_2_O	sym. stretch.	–2.25	–2.23	0.01	0.01	–0.01	–0.01	0.00	0.00
	asym. stretch.	2.78	2.78	–0.04	–0.04	0.02	0.02	–0.01	–0.02
	bend.	–1.22	–1.22	0.80	0.80	0.00	0.00	0.03	0.03
H_2_S	sym. stretch.	1.20	1.20	0.01	0.01	0.01	0.01	0.00	0.00
	asym. stretch.	1.49	1.49	–0.03	–0.03	0.00	0.00	–0.01	–0.01
	bend.	–0.10	–0.10	–0.51	–0.51	–0.01	–0.01	–0.01	–0.01
H_2_Se	sym. stretch.	5.33	5.94	0.04	0.02	–0.05	–0.01	0.00	0.02
	asym. stretch.	7.03	8.51	0.00	0.05	–0.02	–0.10	–0.01	–0.05
	bend.	0.11	0.38	–0.46	–0.46	–0.07	–0.19	–0.01	0.46
H_2_Te	sym. stretch.	–10.88	–12.22	0.04	0.00	–0.07	–0.22	0.04	0.00
	asym. stretch.	–14.43	–19.75	0.02	–0.01	–0.01	0.17	0.02	–0.01
	bend.	–1.05	–2.75	–0.41	–0.02	0.14	0.21	–0.41	–0.02
H_2_Po	sym. stretch.	15.43	–14.57	0.04	–0.01	0.21	0.65	–0.01	0.00
	asym. stretch.	4.49	–26.59	0.03	–0.09	0.01	0.73	0.00	0.00
	bend.	–1.25	–13.42	0.38	0.40	–0.12	–0.47	–0.02	–0.03

a Functional: B3LYP, basis set: aug-cc-pVTZ (on H) + dyall.v3z (on X).

Analysis of the relativistic and nonrelativistic results in Table 2 indicates that the relativistic effects tend to be more pronounced for the derivatives of the coupling constants than for the coupling constants themselves. This is true both for the first and second derivatives of the coupling constants.

When analyzing the results, two interesting observations can be made. First of all, the derivatives with respect to different normal coordinates show different sensitivity to relativity. For instance, in the case of H_2_Te, the relativistic value for  constitutes 262% of the nonrelativistic result, whereas for  it is only 112%. Second, the change from relativistic to nonrelativistic approach can result in significant changes in the derivative, for example, a sign change (e.g. ) or 1 order of magnitude increase of the value (e.g., ).

### ZPV Corrections to Spin–Spin Coupling Constants

The results of the calculations of ZPV corrections to the spin–spin coupling constants computed with both relativistic and nonrelativistic methods for H_2_X, XH_3_, and XH_4_ are presented in Tables 3 and 4 for ^1^*K*_XH_ and ^2^*J*_HH_, respectively.

**Table 3:** ZPV Corrections to ^1^*K*_XH_ [10^19^·m^–2^·kg·s^–2^·Å^–2^] for H_2_X, XH_3_, and XH_4_ Systems Calculated with Relativistic and Nonrelativistic Methods^a^

	nrel			rel		
	harm	anharm	total	harm	anharm	total
H_2_O	0.01	2.98	2.99	0.00	2.98	2.98
H_2_S	–0.55	2.95	2.4	–0.55	2.95	2.4
H_2_Se	–0.77	6.04	5.27	–0.57	7.00	6.43
H_2_Te	–1.79	–10.90	–12.69	–3.03	–14.44	–17.47
H_2_Po	–0.73	–16.99	–17.72	2.75	–25.62	–22.87
NH_3_	–0.13	–3.95	–4.08	–0.13	–4.04	–4.17
PH_3_	–0.54	–1.78	–2.32	–1.00	–2.01	–3.01
AsH_3_	–0.20	–1.03	–1.23	–0.24	–1.20	–1.44
SbH_3_	–3.16	–10.85	–14.01	–0.52	–2.00	–2.52
BiH_3_	–3.02	–15.33	–18.35	–57.76	–85.54	–143.3
CH_4_	0.78	2.64	3.42	0.84	2.71	3.55
SiH_4_	2.00	5.64	7.64	1.94	5.56	7.5
GeH_4_	3.69	6.66	10.35	4.23	7.23	11.46
SnH_4_	5.95	12.90	18.85	7.23	15.28	22.51
PbH_4_	145.09	89.65	124.74	7.66	46.82	54.48

a Functional: B3LYP, basis set: aug-cc-pVTZ (on H) + dyall.v3z (on X).

**Table 4:** ZPV Corrections ^2^*J*_HH_ [Hz] for H_2_X, XH_3_, and XH_4_ Systems Calculated with Relativistic and Nonrelativistic Methods^a^

	nrel			rel		
	harm	anharm	total	harm	anharm	total
H_2_O	0.86	0.13	0.99	0.88	0.12	1.00
H_2_S	–0.86	0.02	–0.84	0.87	–0.02	–0.85
H_2_Se	–1.28	0.15	–1.13	–1.47	0.17	–1.30
H_2_Te	–3.43	–1.65	–5.08	–4.14	–2.13	–6.27
H_2_Po	–1.74	0.31	–1.43	–2.44	–1.00	–3.44
NH_3_	0.28	–0.95	–0.67	0.24	–0.86	–0.62
PH_3_	–1.05	0.43	–0.62	–1.11	0.33	–0.78
AsH_3_	–1.31	0.02	–1.29	–1.54	0.08	–1.46
SbH_3_	–1.60	–0.17	–1.77	–2.01	–0.22	–2.23
BiH_3_	–1.73	0.31	–1.42	–3.25	0.71	–2.54
CH_4_	–0.47	1.34	0.87	–0.5	1.38	0.88
SiH_4_	–0.15	1.08	0.93	–0.18	1.06	0.88
GeH_4_	0.23	1.65	1.88	0.32	2.43	2.75
SnH_4_	0.43	2.44	2.87	0.78	0.46	1.24
PbH_4_	1.82	0.89	2.71	2.27	3.05	5.32

a Functional: B3LYP, basis set: aug-cc-pVTZ (on H) + dyall.v3z (on X).

Because a method for calculating ZPV corrections to NMR parameters is implemented in the Dalton^39,40^ program, some nonrelativistic calculations have been performed with this program in order to check the consistency of the approach. All of the Dalton computations have been run with the same uncontracted basis set and exchange–correlation functional as above. The results can be found in the Supporting Information. In almost all cases, Dalton produces results that are in excellent agreement with the results obtained with our newly implemented method. The only exception is the ZPV correction to ^1^*J*_TeH_, for which the result obtained with Dalton is unphysically large, suggesting a problem with this calculation.

### Effects of Relativity on ^1^*K*_XH_^ZPV^

As shown in Table 3, relativistic effects to the ZPV corrections of ^1^*K*_XH_ become noticeable for lighter systems than was the case for the spin–spin coupling constants themselves. For H_2_Se, PH_3_, AsH_3_, and GeH_4_, the differences between nonrelativistic and relativistic results for the total ZPV correction fall within the range of 10–15% of the relativistic value.

The most striking differences between the ZPV corrections to ^1^*K*_XH_ calculated with nonrelativistic and relativistic approaches occur for SbH_3_, BiH_3_, and PbH_4_. In the case of SbH_3_ and BiH_3_, ^1^*K*_XH_^ZPV^ changes from −14.01 to −2.52 × 10^19^·m^–2^·kg·s^–2^·Å^–2^ and from −18.35 to −143.30 × 10^19^·m^–2^·kg·s^–2^·Å^–2^, respectively. We note that an observed decrease or increase in the value is the same for the spin–spin coupling constant and the corresponding ZPV correction when the method is changed from nonrelativistic to relativistic. The nonrelativistic absolute value of the ZPV correction to the coupling constant for SbH_3_ is larger than the relativistic value of the coupling constant itself, whereas the relativistic value of the ZPV correction constitutes about 20% of the relativistic value of the coupling constant.

An interesting observation can be made for PbH_4_. As the spin–spin coupling constants increase significantly using a relativistic Hamiltonian, the ZPV correction decreases by almost 150%. Furthermore, the nonrelativistic ZPV correction constitutes around 33% of the nonrelativistic coupling constants, whereas this percentage decreases to only 5% for the relativistic results.

In almost all cases, a change in the method from nonrelativistic to relativistic leads to changes in both the harmonic and anharmonic terms that are mostly of the same magnitude, with the two notable exceptions of H_2_Po and PbH_4_. For H_2_Po, the change in the harmonic term is 127%, whereas the change in the anharmonic term is 34%, and for PbH_4_ these changes are 488 and 91%, respectively.

As our main goal is to study relativistic effects on ZPV corrections to spin–spin coupling constants rather than reproduce experimental results, experimental values were not given in Tables 3 and 4. A brief comparison with experimental data in gas phase^41^ and vibrationally averaged reduced spin–spin coupling constants, ^1^*K*_XH_, calculated at the relativistic level is given in Table 5 for CH_4_, SiH_4_, GeH_4_, and SnH_4_. It is clear that in the case of CH_4_ and SiH_4_, adding the ZPV correction does not bring the calculated spin–spin coupling constants closer to experiment. On the other hand, in the case of GeH_4_ and SnH_4_, the agreement becomes much better.

**Table 5:** Comparison of Experimental Values at the Gas phase,^41^^1^*K*_XH_^exp^ [10^19^·m^–2^·kg·s^–2^·Å ^–2^], Calculated Reduced Spin–Spin Coupling Constants at Equilibrium Geometry, ^1^*K*_XH_ [10^19^·m^–2^·kg·s^–2^·Å^–2^], and Vibrationally Averaged Reduced Spin–Spin Coupling Constants, ⟨^1^*K*_XH_⟩ [10^19^·m^–2^·kg·s^–2^·Å^–2^]^a^

	^1^*K*_XH_	⟨^1^*K*_XH_⟩	^1^*K*_XH_^exp^
CH_4_	39.4	43.0	41.4
SiH_4_	80.0	87.5	84.7
GeH_4_	218.6	230.6	232.1
SnH_4_	307.4	361.9	361.9

a Functional: B3LYP, basis set: aug-cc-pVTZ (on H) + dyall.v3z (on X), four-component Dirac–Kohn–Sham Hamiltonian.

### Effects of Relativity on ^2^*J*_HH_^ZPV^

As far as ZPV corrections to ^2^*J*_HH_ are concerned, we in general observe the same trends as for the ZPV corrections to ^1^*J*_XH_. However, it should be noted here that because the values of geminal hydrogen coupling constants are quite small (at most 10 Hz), although the relative changes for the ZPV corrections due to the relativistic effects are quite large, the absolute changes do not exceed a few Hz. We note that for the geminal H–H spin–spin coupling constants, their ZPV corrections are more sensitive to relativistic effects than the couplings themselves in more cases than was the case for the X–H couplings, as this can be seen for H_2_Se, H_2_Te, PH_3_, AsH_3_, SbH_3_, and GeH_4_. Relativistic effects constitute up to 30% of the total value of the ZPV correction to the ^2^*J*_HH_ spin–spin coupling constant in these systems.

As for ^2^*J*_HH_, in almost all cases the relative change in the harmonic and anharmonic terms is of the same magnitude when nonrelativistic and relativistic results are compared, the only exceptions being H_2_Po, AsH_3_, and SnH_4_.

### Effects of Relativity on ZPV Corrections to Spin–Spin Coupling Constants for HC≡CPbH_3_

The results of calculations of spin–spin coupling constants and the corresponding ZPV corrections for HC≡PbH_3_ computed with both relativistic and nonrelativistic methods are given in Table 6. The results are also compared to experimental values.

**Table 6:** Spin–Spin Coupling Constants and Corrections to Coupling Constants for HC≡CPbH_3_ Calculated with Relativistic and Nonrelativistic Methods^a^

	nrel				rel				
		ZPV corr				ZPV corr			
	*J*	harm	anharm	total	*J*	harm	anharm	total	exp^b^
^1^*J*_HC_	228.8	6.99	7.03	14.02	227.6	6.23	6.96	13.19	230^43^ (237.3)
^2^*J*_HC_	47.4	–0.40	0.26	–0.14	42.5	–0.28	0.82	0.54	40.5^43^ (43.3)
^1^*J*_CC_	137.3	–4.24	–2.25	–6.49	142.9	–3.79	–0.65	–4.44	113.0^43^ (125.6)
^1^*J*_CPb_	473.4	–11.97	–42.68	–54.65	245.7	–5.57	–16.37	–21.94	312^c^^,^^42^ (521.8)
									361.5^d^^,^^42^ (571.3)
^2^*J*_CPb_	129.7	–5.69	–3.16	–8.85	105.6	–2.37	–5.72	–8.09	68.0^c^^,^^42^ (123.5)
									75.5^d^^,^^42^ (131.0)
^3^*J*_PbH_	14.1	1.18	6.96	8.14	32.8	–0.96	3.02	2.05	

a Functional: B3LYP, basis set: aug-cc-pVTZ (on H) + dyall.v3z (on X).

b Experimental values for HC≡CPb(C_2_H_5_)_3_, estimated^12^ experimental values for HC≡CPbH_3_ in parenthesis.

c In C_6_D_6_.

d In CDCl_3_.

As far as the comparison of relativistic and nonrelativistic values of the spin–spin coupling constants is concerned, not surprisingly, relativistic effects play a key role in the case of ^1^*J*_CPb_, ^2^*J*_CPb_, and ^3^*J*_PbH_. The HALA effect is almost non-existent for ^1^*J*_HC_ and ^1^*J*_CC_, whereas ^2^*J*_HC_ (geminal coupling with the Pb atom in the middle) decreases by over 10% when a relativistic approach is used.

Using a relativistic Hamiltonian in the calculations of ZPV corrections turns out to be important both for spin–spin coupling constants that involve and do not involve a heavy atom. Relativistic effects constitute from 6% (for ^1^*J*_HC_) to as much as 297% (for ^3^*J*_PbH_) of the total relativistic ZPV correction. An interesting observation can be made for the ZPV correction to ^2^*J*_CPb_. Even though the differences between the total ZPV corrections calculated with relativistic and nonrelativistic methods are relatively small, the changes of harmonic and anharmonic contributions are much larger. The harmonic contribution increases and the anharmonic contribution decreases and these changes partially cancel each other in the total value of the ZPV correction. The cancellation of the relativistic effect is thus coincidental, and in other cases, the ZPV corrections on the one-bond couplings of this type may be much more affected by relativity, as seen for the H_2_X, XH_3_ and XH_4_ systems.

The available experimental data refer to the ethylene-substituted acetylene derivative HC≡CPb(C_2_H_5_)_3_, whereas the coupling constants and ZPV corrections discussed below have been calculated for compounds containing hydrogen atoms instead of ethylene groups. In ref (12), the influence of such a substitution was studied and a correction to the experimental value for HC≡CPb(C_2_H_5_)_3_ can be introduced so as to estimate an “experimental” value for HC≡CPbH_3_. These values are given in parentheses next to the experimental values for HC≡CPb(C_2_H_5_)_3_ in Table 6. It can be noticed that for ^1^*J*_HC_, ^2^*J*_HC_ and ^1^*J*_CC_, adding the ZPV calculated using a relativistic approach brings the spin–spin coupling constants closer to the estimated “experimental” value, whereas for ^1^*J*_CPb_ and ^2^*J*_CPb_ the ZPV correction brings the calculated coupling constant further from the estimated “experimental” value. However, the vibrational effects are not the only effects that should be taken into account when comparing computational results to experiment. A study of available experimental data shows that in this case, solvent effects might also play an important role.^42^ Moreover, the remaining disagreement with experiment might also be due to the errors resulting from the use of DFT with the B3LYP functional.

## Conclusions

We have presented a numerical method for calculating the ZPV corrections to spin–spin coupling constants with relativistic four-component DFT. Test calculations have been performed for hydrides of elements from groups 14, 15, and 16, and for HC≡CPbH_3_ in order to demonstrate the versatility of the method.

For both the ZPV corrections to spin–spin coupling constants and the derivatives of the spin–spin coupling constants, the effects of relativity become notable much earlier in terms of the atomic number of the heavy element, for example selenium and germanium, compared to the spin–spin coupling constants. Moreover, our calculations demonstrate that as far as molecules containing heavier atoms are concerned, for instance BiH_3_ and PbH_4_, relativistic effects have such a great impact on the results that the commonly used scheme in which ZPV corrections are calculated using a nonrelativistic Hamiltonian and added to the relativistic values, simply cannot be considered reliable.

In addition to this, ZPV corrections to spin–spin coupling constants have been computed for HC≡CPbH_3_. Relativistic effects turned out to be at least noticeable, if not crucial, for all the calculated ZPV corrections to spin–spin coupling constants. Analysis of the results obtained shows that relativity should be taken into account for couplings that involve a heavy atom.

## Supporting Information Available

Comparison of results for ZPV corrections to spin–spin coupling constants calculated with Dalton and our newly implemented method and different optimized geometries (PDF)
