# Supplementary material for: Vibrational Corrections to NMR Spin–Spin Coupling Constants from Relativistic Four-Component DFT Calculations
Source: J Phys Chem A. 2022 Sep 22;126(39):7013–20. doi: 10.1021/acs.jpca.2c05019 (PMC9549459; doi:10.1021/acs.jpca.2c05019)
Supplement: Supplementary file 1 — jp2c05019_si_001.pdf [file jp2c05019_si_001.pdf]

Vibrational Corrections to NMR Spin-Spin Coupling Constants from Relativistic Four-Component DFT Calculations

Supporting Information

Katarzyna Jakubowska,[†] Magdalena Pecul,[*,†] and Kenneth Ruud[‡,¶]

† *Faculty of Chemistry, University of Warsaw, Warsaw, Poland*

‡*Hylleraas Centre for Quantum Molecular Sciences, Department of Chemistry, UiT – The Arctic University of Norway, N-9037 Tromsø, Norway*

¶*Norwegian Defence Research Establishment, P.O. Box 25, 2027 Kjeller, Norway*

E-mail: mpecul@chem.uw.edu.pl

Supporting Information

Comparison of results for ZPV corrections to spin-spin coupling constants calculated with DALTON and our newly implemented method

Table S1: ZPV corrections to $^{1}J_{XH}$ [Hz] for H_2X systems calculated with DALTON and our newly implemented method at nonrelativistic approach[a]

	DALTON			This work		
	harm	anharm	total	harm	anharm	total
H_2O	-0.03	4.85	-4.88	-0.02	4.85	-4.87
H_2S	-0.53	-2.69	2.16	-0.5	-2.68	2.18
H_2Se	-3.22	-13.95	10.73	-1.78	-14.05	12.27
H_2Te	389.98	-348.89	738.87	6.87	-41.78	48.65

[a] functional:B3LYP, basis set: aug-cc-pVTZ (on H) + dyall.v3z (on X)

Table S2: ZPV corrections to $^{2}J_{HH}$ [Hz] for H_2X systems calculated with DALTON and our newly implemented method at nonrelativistic approach[a]

	DALTON			This work		
	harm	anharm	total	harm	anharm	total
H_2O	0.84	-0.17	1.00	0.86	-0.13	0.99
H_2S	-0.86	-0.01	-0.85	-0.86	-0.02	-0.84
H_2Se	-1.33	-0.12	-1.21	-1.28	-0.15	-1.13
H_2Te	-3.57	1.63	-5.21	-3.43	1.65	-5.08

[a] functional:B3LYP, basis set: aug-cc-pVTZ (on H) + dyall.v3z (on X)

Optimized Geometries

All geometry optimization calculations have been carried out with the DIRAC program. The uncontracted aug-cc-pVDZ basis set on the hydrogen atoms and the uncontracted triple-ζ Dyall's basis set17–19 (dyall.v3z) on all the other atoms have been applied together with the B3LYP exchange-correlation functional. The xyz coordinates are given in Angstrom.

H_2O - rel

H	0.9613133760	0.0000000000	0.0052918765
H	-0.2435436859	0.0000000000	-0.9299677918
O	0.0009803099	0.0000000000	-0.0012640847

H_2O - nonrel

H	0.9621531629	0.0000000000	0.0055985260
H	-0.2440490289	0.0000000000	-0.9307052822
O	0.0006458659	0.0000000000	-0.0008332438

H_2S - rel

H	1.1973804691	0.0000000000	0.0421453195
H	-0.3377929144	0.0000000000	-1.1495193477
S	-0.1408375547	0.0000000000	0.1814340282

H_2S - nonrel

H	1.1977547409	0.0000000000	0.0427881492
H	-0.3385084883	0.0000000000	-1.1497225287
S	-0.1404962525	0.0000000000	0.1809943795

H_2Se - rel

H	1.2781237820	0.0000000000	0.0628855527
H	-0.3779103141	0.0000000000	-1.2225960196
Se	-0.1814634679	0.0000000000	0.2337704669

H_2Se - nonrel

H	1.2805785698	0.0000000000	0.0655273421
H	-0.3810783845	0.0000000000	-1.2243189485
Se	-0.1807501853	0.0000000000	0.2328516064

H_2Te - rel

H	1.4139170141	0.0000000000	0.1070368483
H	-0.4543602670	0.0000000000	-1.3431963489
Te	-0.2408067471	0.0000000000	0.3102195006

H_2Te - nonrel

H	1.4196308146	0.0000000000	0.1128000416
H	-0.4613604131	0.0000000000	-1.3473020230
Te	-0.2395204015	0.0000000000	0.3085619815

H_2Po - rel

H	1.4819462192	0.0000000000	0.1250917157
H	-0.4887263171	0.0000000000	-1.4046357492
Po	-0.2744699021	0.0000000000	0.3536040336

H_2Po - nonrel

H	1.4859317719	0.0000000000	0.1352246061
H	-0.4995289609	0.0000000000	-1.4059673977
Po	-0.2676528111	0.0000000000	0.3448027917

NH_3 - rel

H	0.2689263565	0.6559375500	0.6865885359
H	0.7845316060	-0.4553946229	-0.3888170611
H	-0.5554421872	0.4193698339	-0.6997226045
N	-0.0423157753	-0.0527127610	0.0341511296

NH_3 - nonrel

H	0.2690999909	0.6564343159	0.6872422253
H	0.7851574718	-0.4558726549	-0.3891068237
H	-0.5559933506	0.4196593049	-0.7002861811
N	-0.0425641121	-0.0530209658	0.0343507795

PH_3 - rel

H	0.3522882298	0.8463985907	0.8631579655
H	1.0070480256	-0.5648499152	-0.5024551079
H	-0.6945485886	0.5459919306	-0.8972569413
P	-0.2090876667	-0.2603406061	0.1687540837

PH_3 - nonrel

H	0.3521795690	0.8465400113	0.8640247231
H	1.0073838528	-0.5656664767	-0.5025152739
H	-0.6953681196	0.5459295978	-0.8975853614
P	-0.2084953023	-0.2596031324	0.1682759121

AsH_3 - rel

H	0.3709463449	0.8930541379	0.9139797468
H	1.0633029099	-0.5992300344	-0.5300476568
H	-0.7359863852	0.5753943153	-0.9475088950
As	-0.2425628695	-0.3020184188	0.1957768050

AsH_3 - nonrel

H	0.3708736857	0.8946264584	0.9187193502
H	1.0659014458	-0.6034140987	-0.5308781522
H	-0.7403303691	0.5757420013	-0.9499507284
As	-0.2407447624	-0.2997543610	0.1943095304

SbH_3 - rel

H	0.4042547589	0.9876606199	1.0365246093
H	1.1818910252	-0.6884297271	-0.5853618359
H	-0.8389844346	0.6308670731	-1.0542156139
Sb	-0.2914613495	-0.3628979659	0.2352528405

SbH_3 - nonrel

H	0.4045448841	0.9921864950	1.0480893152
H	1.1889060127	-0.6983910201	-0.5878130742
H	-0.8494801884	0.6323190542	-1.0607455025
Sb	-0.2882707084	-0.3589145291	0.2326692615

BiH_3 - rel

H	0.4183192084	1.0244482471	1.0795092004
H	1.2267607849	-0.7183283455	-0.6070020535
H	-0.8743754082	0.6533515478	-1.0945250915
Bi	-0.3150045851	-0.3922714494	0.2542179446

BiH_3 - nonrel

H	0.4203548129	1.0378388567	1.1082965304
H	1.2463970726	-0.7425304575	-0.6144814694
H	-0.9002371319	0.6588674701	-1.1125046480
Bi	-0.3108147536	-0.3869758693	0.2508895871

CH_4 - rel

H	0.5290456846	0.1610914326	0.9363179348
H	0.2052132398	0.8243849488	-0.6788912803
H	0.3346592668	-0.9318368913	-0.4498076586
H	-1.0690087892	-0.0537287163	0.1922011265
C	-0.0000094021	-0.0000107738	-0.0000201225

CH_4 - nonrel

H	0.5295313847	0.1612517088	0.9371711176
H	0.2054201616	0.8251594756	-0.6795051003
H	0.3349768055	-0.9327110957	-0.4502224335
H	-1.0700089208	-0.0537806251	0.1923951063
C	-0.0000194310	-0.0000194637	-0.0000386901

SiH_4 - rel

H	0.7212918080	0.2196372267	1.2765594029
H	0.2797949993	1.1239518614	-0.9255611272
H	0.4562786617	-1.2704346765	-0.6132395661
H	-1.4574551669	-0.0732430177	0.2620639188
Si	-0.0000103021	-0.0000113940	-0.0000226284

SiH_4 - nonrel

H	0.7215406224	0.2197122929	1.2770006537
H	0.2798906329	1.1243391193	-0.9258814092
H	0.4564355832	-1.2708723339	-0.6134511604
H	-1.4579571045	-0.0732682243	0.2621536436
Si	-0.0000097340	-0.0000108540	-0.0000217277

GeH_4 - rel

H	0.7430784916	0.2262535813	1.3151262809
H	0.2882221762	1.1578733486	-0.9535272542
H	0.4700455361	-1.3087763335	-0.6317555339
H	-1.5014504094	-0.0754526447	0.2699575260
Ge	0.0000042055	0.0000020483	-0.0000010188

GeH_4 - nonrel

H	0.7462420643	0.2272167730	1.3207260028
H	0.2894491007	1.1628030606	-0.9575870742
H	0.4720466757	-1.3143482641	-0.6344451065

H	-1.5078420915	-0.0757737341	0.2711069500
Ge	0.0000042508	0.0000021646	-0.0000007721

SnH_4 - rel

H	0.8328683414	0.2535858909	1.4740439583
H	0.3230383437	1.2977683403	-1.0687428186
H	0.5268363788	-1.4668987516	-0.7080835835
H	-1.6828567169	-0.0845657720	0.3025700373
Sn	0.0000136531	0.0000102924	0.0000124065

SnH_4 - nonrel

H	0.8416794681	0.2562681626	1.4896397431
H	0.3264552364	1.3114986892	-1.0800502886
H	0.5324099720	-1.4824176861	-0.7155742341
H	-1.7006586463	-0.0854599542	0.3057710429
Sn	0.0000139699	0.0000107885	0.0000137367

PbH_4 - rel

H	0.8529454128	0.2596969282	1.5095755218
H	0.3308197381	1.3290355640	-1.0944978318
H	0.5395283085	-1.5022394177	-0.7251459469
H	-1.7234082310	-0.0866038246	0.3098568297
Pb	0.0000147717	0.0000107502	0.0000114272

PbH_4 - nonrel

H	0.8823861447	0.2686621440	1.5616852962
H	0.3422429763	1.3749260848	-1.1322830792

H	0.5581584736	-1.5541089589	-0.7501790714
H	-1.7829035917	-0.0895920144	0.3205594256
Pb	0.0000159970	0.0000127445	0.0000174287
